# 1-Benzyl-2,5-diphenyl-3-tosylimidazol­idin-4-one

**DOI:** 10.1107/S1600536811032284

**Published:** 2011-08-17

**Authors:** K. Sakthimurugesan, S. Ranjith, A. SubbiahPandi, K. Namitharan, K. Pitchumani

**Affiliations:** a Department of Physics, Presidency College (Autonomous), Chennai 600 005, India; b School of Chemistry, Madurai Kamaraj University, Madurai 625 021, India

## Abstract

In the title compound, C_29_H_26_N_2_O_3_S, the central imidazolidine ring adopts an envelope conformation with the N atom bearing the benzyl ring at the flap. The S atom has distorted tetra­hedral geometry. The benzyl and tosyl rings are oriented at a dihedral angle of 52.1 (1)°. The phenyl rings connected to the imidazolidine ring form a dihedral angle of 28.7 (1)°.

## Related literature

For the biological activity of sulfonamides, see: Zareef *et al.* (2007 ▶); Chohan & Shad (2007 ▶); Pomarnacka & Kozlarska-Kedra (2003 ▶); Nieto *et al.* (2005 ▶); Wang *et al.* (1995 ▶). For a related structure, see: Ranjith *et al.* (2011 ▶). For puckering parameters, see: Cremer & Pople (1975 ▶). For asymmetry parameters, see: Nardelli *et al.* (1983 ▶).
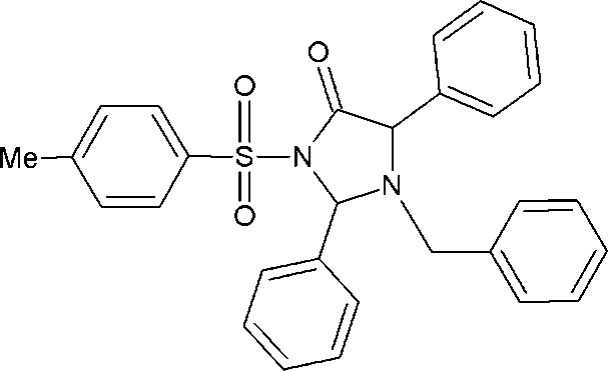


## Experimental

### 

#### Crystal data

C_29_H_26_N_2_O_3_S
*M* *_r_* = 482.58
Monoclinic, 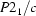

*a* = 18.6024 (7) Å
*b* = 8.0489 (3) Å
*c* = 17.0860 (6) Å
β = 106.426 (2)°
*V* = 2453.85 (16) Å^3^
*Z* = 4
Mo *K*α radiation
μ = 0.17 mm^−1^
*T* = 293 K
0.25 × 0.22 × 0.19 mm

#### Data collection

Bruker APEXII CCD area-detector diffractometer
Absorption correction: multi-scan (*SADABS*; Sheldrick, 1996 ▶) *T*_min_ = 0.981, *T*_max_ = 0.985
25449 measured reflections
4805 independent reflections
3598 reflections with *I* > 2σ(*I*)
*R*_int_ = 0.032

#### Refinement

*R*[*F* ^2^ > 2σ(*F* ^2^)] = 0.039
*wR*(*F* ^2^) = 0.115
*S* = 1.01
4805 reflections
317 parameters
H-atom parameters constrained
Δρ_max_ = 0.15 e Å^−3^
Δρ_min_ = −0.38 e Å^−3^

### 

Data collection: *APEX2* (Bruker, 2004 ▶); cell refinement: *SAINT* (Bruker, 2004 ▶); data reduction: *SAINT*; program(s) used to solve structure: *SHELXS97* (Sheldrick, 2008 ▶); program(s) used to refine structure: *SHELXL97* (Sheldrick, 2008 ▶); molecular graphics: *ORTEP-3* (Farrugia, 1997 ▶); software used to prepare material for publication: *SHELXL97* and *PLATON* (Spek, 2009 ▶).

## Supplementary Material

Crystal structure: contains datablock(s) global, I. DOI: 10.1107/S1600536811032284/ci5194sup1.cif

Structure factors: contains datablock(s) I. DOI: 10.1107/S1600536811032284/ci5194Isup2.hkl

Supplementary material file. DOI: 10.1107/S1600536811032284/ci5194Isup3.cml

Additional supplementary materials:  crystallographic information; 3D view; checkCIF report

## Figures and Tables

**Fig. 1.**
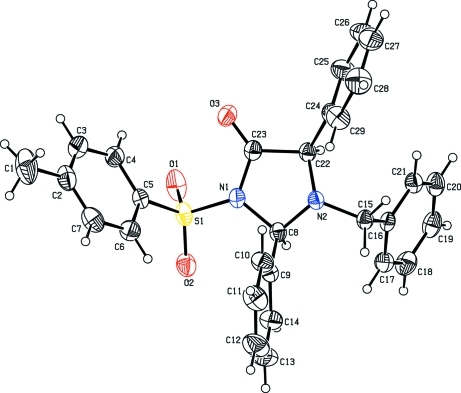
The molecular structure of the title compound, showing the atom-numbering scheme. Displacement ellipsoids are drawn at the 30% probability level.

## supplementary crystallographic information

### Comment

Sulfonamides have widely been recognized for their wide variety of pharmacological activities such as antibacterial, antitumor, anti-carbonic anhydrase, diuretic, hypoglycaemic, antithyroid and protease inhibitory activity. Sulfonamides, particularly sulfadiazine and sulfadoxine, have also been used clinically as antimalarial agents (Zareef *et al.*, 2007). Due to their significant pharmacological applications and widespread use in medicine, these compounds have also gained attention in bioinorganic and metal-based drug chemistry (Chohan *et al.*, 2007). Sulfonamide derivatives are well known drugs and are used to control diseases caused by bacterial infections. Benzene sulfonamide derivatives are known to exhibit anticancer and HIV activities (Pomarnacka & Kozlarska-Kedra, 2003) and antibacterial activities (Nieto *et al.*, 2005). Imidazolidine compounds are important intermediates and building blocks in the construction of various biologically active compounds (Wang *et al.*, 1995). Against this background, and in order to obtain detailed information on molecular conformations in the solid state, an X-ray study of the title compound was carried out.

X-Ray analysis confirms the molecular structure and atom connectivity as illustrated in Fig. 1. The geometry around the S atom is distorted tetrahedral, with a O1—S1—O2 angle of 120.5 (1)°. The widening of this angle may be due to repulsive interactions between the two short S═O bonds, similar to that observed in a related structure (Ranjith *et al.*, 2011). The S—O, S—C and S–N distances are comparable to those observed in similar structures (Ranjith *et al.*, 2011). The methyl atom C1 deviates by 0.021 (3) Å from the plane of the C2–C7 ring.

The imidazolidine ring adopts an envelope conformation, with the puckering parameters q_2_ and φ (Cremer & Pople, 1975) and the smallest displacement asymmetric parameters, Δ, (Nardelli *et al.*, 1983) as follows: q_2_ = 0.302 (2) Å, φ = 257.1 (3)° and Δ_s_(N2) = 3.3 (2)°. The methylbenzene ring (C2–C7) makes dihedral angles of 43.6 (1), 52.1 (1) and 72.3 (1)° with respect to the C9–C14, C16–C21 and C24–C29 benzene rings.

The molecules lack hydrogen bonding functionality. The crystal packing is stabilized by van der Waals interactions.

### Experimental

Alkyne (1 mmol) in dichloromethane (1 ml) was added slowly to a mixture of CuI-zeolite (30 mg), 4-toluene sulfonyl azide (1 mmol), *N*-benzylnitrone (1 mmol) and triethylamine (1.2 mmol) in dichloromethane (2 ml) under N_2_ atmosphere. After stirring at room temperature for the 3 h, the mixture was diluted with dichloromethane. After removing the catalyst by filtration, followed by solvent evaporation under reduced pressure, the resulting crude product was finally purified by column chromatography on silica gel (60–120 mesh) with ethyl acetate and petroleum ether as eluting solvent to give the desired product. Single crystals suitable for X-ray diffraction were obtained by slow evaporation of a solution of the title compound in acetone at room temperature.

### Refinement

H atoms were positioned geometrically [C–H = 0.93–0.97 Å] and allowed to ride on their parent C atoms, with *U*_iso_(H) = 1.2*U*_eq_(C) [1.5*U*_eq_(C) for methyl H atoms].

### Crystal data

**Table:**

C_29_H_26_N_2_O_3_S	*F*(000) = 1016
*M**_r_* = 482.58	*D*_x_ = 1.306 Mg m^−^^3^
Monoclinic, *P*2_1_/*c*	Mo *K*α radiation, λ = 0.71073 Å
Hall symbol: -P 2ybc	Cell parameters from 4805 reflections
*a* = 18.6024 (7) Å	θ = 1.1–26.0°
*b* = 8.0489 (3) Å	µ = 0.17 mm^−^^1^
*c* = 17.0860 (6) Å	*T* = 293 K
β = 106.426 (2)°	Block, white crystalline
*V* = 2453.85 (16) Å^3^	0.25 × 0.22 × 0.19 mm
*Z* = 4	

### Data collection

**Table:**

Bruker APEXII CCD area-detector diffractometer	4805 independent reflections
Radiation source: fine-focus sealed tube	3598 reflections with *I* > 2σ(*I*)
graphite	*R*_int_ = 0.032
ω and φ scans	θ_max_ = 26.0°, θ_min_ = 1.1°
Absorption correction: multi-scan (*SADABS*; Sheldrick, 1996)	*h* = −22→20
*T*_min_ = 0.981, *T*_max_ = 0.985	*k* = −9→8
25449 measured reflections	*l* = −20→21

### Refinement

**Table:**

Refinement on *F*^2^	Primary atom site location: structure-invariant direct methods
Least-squares matrix: full	Secondary atom site location: difference Fourier map
*R*[*F*^2^ > 2σ(*F*^2^)] = 0.039	Hydrogen site location: inferred from neighbouring sites
*wR*(*F*^2^) = 0.115	H-atom parameters constrained
*S* = 1.01	*w* = 1/[σ^2^(*F*_o_^2^) + (0.0556*P*)^2^ + 0.6284*P*] where *P* = (*F*_o_^2^ + 2*F*_c_^2^)/3
4805 reflections	(Δ/σ)_max_ = 0.001
317 parameters	Δρ_max_ = 0.15 e Å^−^^3^
0 restraints	Δρ_min_ = −0.38 e Å^−^^3^

### Special details

**Table:**

Geometry. All e.s.d.'s (except the e.s.d. in the dihedral angle between two l.s. planes) are estimated using the full covariance matrix. The cell e.s.d.'s are taken into account individually in the estimation of e.s.d.'s in distances, angles and torsion angles; correlations between e.s.d.'s in cell parameters are only used when they are defined by crystal symmetry. An approximate (isotropic) treatment of cell e.s.d.'s is used for estimating e.s.d.'s involving l.s. planes.
Refinement. Refinement of *F*^2^ against ALL reflections. The weighted *R*-factor *wR* and goodness of fit *S* are based on *F*^2^, conventional *R*-factors *R* are based on *F*, with *F* set to zero for negative *F*^2^. The threshold expression of *F*^2^ > σ(*F*^2^) is used only for calculating *R*-factors(gt) *etc*. and is not relevant to the choice of reflections for refinement. *R*-factors based on *F*^2^ are statistically about twice as large as those based on *F*, and *R*- factors based on ALL data will be even larger.

### Fractional atomic coordinates and isotropic or equivalent isotropic displacement parameters (Å^2^)

**Table:**

	*x*	*y*	*z*	*U*_iso_*/*U*_eq_	
C1	0.59892 (15)	1.1019 (4)	0.3124 (2)	0.1139 (11)	
H1A	0.6361	1.1075	0.3644	0.171*	
H1B	0.5989	1.2039	0.2833	0.171*	
H1C	0.6103	1.0111	0.2814	0.171*	
C2	0.52227 (12)	1.0751 (3)	0.32515 (14)	0.0663 (6)	
C3	0.45945 (13)	1.1508 (3)	0.27587 (14)	0.0714 (6)	
H3	0.4644	1.2209	0.2344	0.086*	
C4	0.38964 (11)	1.1257 (3)	0.28613 (12)	0.0584 (5)	
H4	0.3478	1.1777	0.2519	0.070*	
C5	0.38237 (9)	1.0228 (2)	0.34764 (10)	0.0421 (4)	
C6	0.44422 (10)	0.9453 (2)	0.39808 (12)	0.0548 (5)	
H6	0.4393	0.8750	0.4395	0.066*	
C7	0.51360 (11)	0.9735 (3)	0.38628 (14)	0.0665 (6)	
H7	0.5556	0.9223	0.4207	0.080*	
C8	0.23093 (9)	0.6933 (2)	0.36626 (10)	0.0414 (4)	
H8	0.1928	0.7370	0.3904	0.050*	
C9	0.29247 (9)	0.6072 (2)	0.43025 (10)	0.0453 (4)	
C10	0.34796 (11)	0.5227 (3)	0.40738 (12)	0.0576 (5)	
H10	0.3475	0.5206	0.3528	0.069*	
C11	0.40383 (12)	0.4417 (3)	0.46466 (15)	0.0739 (6)	
H11	0.4412	0.3853	0.4491	0.089*	
C12	0.40401 (15)	0.4446 (3)	0.54519 (15)	0.0843 (7)	
H12	0.4423	0.3919	0.5843	0.101*	
C13	0.34830 (16)	0.5244 (3)	0.56805 (14)	0.0809 (7)	
H13	0.3480	0.5234	0.6224	0.097*	
C14	0.29260 (12)	0.6062 (3)	0.51072 (11)	0.0614 (5)	
H14	0.2549	0.6611	0.5265	0.074*	
C15	0.14866 (9)	0.4518 (2)	0.31824 (11)	0.0435 (4)	
H15A	0.1747	0.4021	0.3702	0.052*	
H15B	0.1417	0.3655	0.2771	0.052*	
C16	0.07241 (9)	0.50676 (19)	0.32246 (10)	0.0405 (4)	
C17	0.05947 (11)	0.5553 (2)	0.39499 (12)	0.0544 (5)	
H17	0.0989	0.5565	0.4427	0.065*	
C18	−0.01143 (12)	0.6018 (3)	0.39741 (14)	0.0649 (6)	
H18	−0.0192	0.6350	0.4465	0.078*	
C19	−0.07037 (11)	0.5994 (2)	0.32768 (14)	0.0617 (5)	
H19	−0.1181	0.6298	0.3295	0.074*	
C20	−0.05838 (10)	0.5521 (2)	0.25558 (13)	0.0580 (5)	
H20	−0.0981	0.5513	0.2081	0.070*	
C21	0.01189 (10)	0.5057 (2)	0.25284 (12)	0.0489 (4)	
H21	0.0191	0.4730	0.2034	0.059*	
C22	0.16901 (9)	0.6743 (2)	0.22488 (10)	0.0419 (4)	
H22	0.1195	0.7190	0.2229	0.050*	
C23	0.22466 (9)	0.8161 (2)	0.23794 (11)	0.0455 (4)	
C24	0.16290 (10)	0.5747 (2)	0.14846 (10)	0.0451 (4)	
C25	0.11035 (13)	0.6192 (3)	0.07717 (12)	0.0692 (6)	
H25	0.0789	0.7093	0.0766	0.083*	
C26	0.10414 (17)	0.5299 (4)	0.00611 (14)	0.0879 (8)	
H26	0.0685	0.5605	−0.0419	0.105*	
C27	0.14972 (16)	0.3984 (3)	0.00633 (15)	0.0830 (7)	
H27	0.1455	0.3393	−0.0415	0.100*	
C28	0.20162 (15)	0.3530 (3)	0.07650 (16)	0.0812 (7)	
H28	0.2328	0.2627	0.0766	0.097*	
C29	0.20811 (12)	0.4407 (3)	0.14747 (13)	0.0638 (5)	
H29	0.2436	0.4086	0.1953	0.077*	
N1	0.25830 (8)	0.82623 (17)	0.32174 (8)	0.0452 (3)	
N2	0.19719 (7)	0.58031 (16)	0.29956 (8)	0.0404 (3)	
O1	0.24894 (7)	1.13491 (17)	0.32774 (10)	0.0749 (5)	
O2	0.30718 (8)	0.97342 (18)	0.45167 (8)	0.0668 (4)	
O3	0.23762 (7)	0.90679 (16)	0.18789 (8)	0.0610 (4)	
S1	0.29541 (2)	1.00278 (5)	0.36717 (3)	0.04955 (15)	

### Atomic displacement parameters (Å^2^)

**Table:**

	*U*^11^	*U*^22^	*U*^33^	*U*^12^	*U*^13^	*U*^23^
C1	0.0717 (17)	0.137 (3)	0.153 (3)	−0.0412 (18)	0.0645 (18)	−0.059 (2)
C2	0.0541 (13)	0.0747 (14)	0.0762 (14)	−0.0185 (11)	0.0283 (11)	−0.0283 (12)
C3	0.0761 (16)	0.0759 (15)	0.0662 (13)	−0.0238 (13)	0.0269 (12)	0.0004 (11)
C4	0.0532 (12)	0.0566 (11)	0.0588 (11)	−0.0067 (10)	0.0050 (9)	0.0084 (9)
C5	0.0362 (9)	0.0389 (9)	0.0485 (9)	−0.0027 (7)	0.0077 (7)	−0.0069 (7)
C6	0.0466 (11)	0.0569 (11)	0.0574 (11)	0.0054 (9)	0.0092 (9)	0.0047 (9)
C7	0.0393 (11)	0.0765 (15)	0.0769 (14)	0.0085 (10)	0.0051 (10)	−0.0123 (12)
C8	0.0348 (9)	0.0426 (9)	0.0487 (9)	−0.0014 (7)	0.0146 (7)	−0.0042 (7)
C9	0.0392 (9)	0.0472 (10)	0.0470 (9)	−0.0033 (8)	0.0082 (7)	−0.0025 (8)
C10	0.0475 (11)	0.0686 (13)	0.0538 (11)	0.0074 (10)	0.0096 (9)	−0.0021 (9)
C11	0.0531 (13)	0.0760 (15)	0.0832 (16)	0.0162 (11)	0.0038 (11)	−0.0034 (12)
C12	0.0822 (18)	0.0794 (16)	0.0699 (15)	0.0139 (14)	−0.0134 (13)	0.0098 (13)
C13	0.0952 (19)	0.0859 (17)	0.0519 (12)	0.0079 (15)	0.0053 (12)	0.0054 (11)
C14	0.0642 (13)	0.0693 (13)	0.0499 (11)	0.0030 (11)	0.0149 (9)	−0.0016 (9)
C15	0.0391 (9)	0.0359 (8)	0.0534 (10)	−0.0006 (7)	0.0097 (8)	0.0023 (7)
C16	0.0370 (9)	0.0321 (8)	0.0520 (9)	−0.0033 (7)	0.0120 (7)	0.0029 (7)
C17	0.0485 (11)	0.0609 (11)	0.0540 (11)	−0.0055 (9)	0.0146 (9)	0.0009 (9)
C18	0.0650 (14)	0.0648 (13)	0.0769 (14)	−0.0025 (11)	0.0398 (12)	−0.0036 (11)
C19	0.0440 (11)	0.0486 (11)	0.0983 (16)	0.0030 (9)	0.0293 (11)	0.0086 (11)
C20	0.0384 (10)	0.0512 (11)	0.0783 (14)	−0.0009 (9)	0.0064 (9)	0.0082 (10)
C21	0.0425 (10)	0.0461 (10)	0.0562 (10)	−0.0024 (8)	0.0109 (8)	−0.0001 (8)
C22	0.0349 (9)	0.0414 (9)	0.0485 (9)	0.0004 (7)	0.0104 (7)	0.0013 (7)
C23	0.0390 (9)	0.0422 (9)	0.0546 (10)	0.0013 (8)	0.0119 (8)	0.0022 (8)
C24	0.0425 (10)	0.0457 (10)	0.0483 (9)	−0.0075 (8)	0.0147 (8)	−0.0016 (8)
C25	0.0835 (16)	0.0604 (13)	0.0554 (12)	0.0079 (12)	0.0060 (11)	−0.0002 (10)
C26	0.114 (2)	0.0915 (19)	0.0479 (12)	−0.0014 (16)	0.0059 (13)	−0.0045 (12)
C27	0.110 (2)	0.0846 (18)	0.0618 (14)	−0.0159 (16)	0.0372 (14)	−0.0192 (13)
C28	0.0861 (17)	0.0805 (16)	0.0884 (17)	0.0104 (14)	0.0433 (14)	−0.0187 (13)
C29	0.0532 (12)	0.0716 (13)	0.0679 (13)	0.0111 (11)	0.0192 (10)	−0.0088 (11)
N1	0.0424 (8)	0.0405 (8)	0.0512 (8)	−0.0072 (6)	0.0109 (6)	−0.0030 (6)
N2	0.0346 (7)	0.0390 (7)	0.0455 (7)	−0.0026 (6)	0.0079 (6)	−0.0006 (6)
O1	0.0429 (8)	0.0440 (8)	0.1284 (13)	0.0088 (6)	0.0087 (8)	−0.0106 (8)
O2	0.0708 (10)	0.0728 (9)	0.0662 (9)	−0.0216 (8)	0.0346 (7)	−0.0267 (7)
O3	0.0617 (9)	0.0556 (8)	0.0642 (8)	−0.0102 (7)	0.0153 (7)	0.0122 (7)
S1	0.0380 (3)	0.0413 (3)	0.0695 (3)	−0.00206 (19)	0.0156 (2)	−0.0123 (2)

### Geometric parameters (Å, °)

**Table:**

C1—C2	1.517 (3)	C15—H15B	0.97
C1—H1A	0.96	C16—C17	1.384 (2)
C1—H1B	0.96	C16—C21	1.388 (2)
C1—H1C	0.96	C17—C18	1.383 (3)
C2—C7	1.372 (3)	C17—H17	0.93
C2—C3	1.375 (3)	C18—C19	1.372 (3)
C3—C4	1.374 (3)	C18—H18	0.93
C3—H3	0.93	C19—C20	1.367 (3)
C4—C5	1.375 (2)	C19—H19	0.93
C4—H4	0.93	C20—C21	1.373 (3)
C5—C6	1.377 (2)	C20—H20	0.93
C5—S1	1.7492 (17)	C21—H21	0.93
C6—C7	1.380 (3)	C22—N2	1.448 (2)
C6—H6	0.93	C22—C24	1.509 (2)
C7—H7	0.93	C22—C23	1.514 (2)
C8—N2	1.454 (2)	C22—H22	0.98
C8—N1	1.484 (2)	C23—O3	1.199 (2)
C8—C9	1.510 (2)	C23—N1	1.393 (2)
C8—H8	0.98	C24—C29	1.370 (3)
C9—C14	1.374 (2)	C24—C25	1.376 (3)
C9—C10	1.382 (3)	C25—C26	1.387 (3)
C10—C11	1.374 (3)	C25—H25	0.93
C10—H10	0.93	C26—C27	1.356 (4)
C11—C12	1.375 (3)	C26—H26	0.93
C11—H11	0.93	C27—C28	1.360 (3)
C12—C13	1.367 (4)	C27—H27	0.93
C12—H12	0.93	C28—C29	1.379 (3)
C13—C14	1.376 (3)	C28—H28	0.93
C13—H13	0.93	C29—H29	0.93
C14—H14	0.93	N1—S1	1.6719 (14)
C15—N2	1.466 (2)	O1—S1	1.4152 (14)
C15—C16	1.507 (2)	O2—S1	1.4176 (15)
C15—H15A	0.97		
			
C2—C1—H1A	109.5	C21—C16—C15	120.25 (15)
C2—C1—H1B	109.5	C18—C17—C16	120.81 (18)
H1A—C1—H1B	109.5	C18—C17—H17	119.6
C2—C1—H1C	109.5	C16—C17—H17	119.6
H1A—C1—H1C	109.5	C19—C18—C17	120.31 (19)
H1B—C1—H1C	109.5	C19—C18—H18	119.8
C7—C2—C3	117.91 (19)	C17—C18—H18	119.8
C7—C2—C1	120.8 (2)	C20—C19—C18	119.51 (18)
C3—C2—C1	121.3 (2)	C20—C19—H19	120.2
C4—C3—C2	121.8 (2)	C18—C19—H19	120.2
C4—C3—H3	119.1	C19—C20—C21	120.41 (19)
C2—C3—H3	119.1	C19—C20—H20	119.8
C3—C4—C5	119.14 (19)	C21—C20—H20	119.8
C3—C4—H4	120.4	C20—C21—C16	121.24 (18)
C5—C4—H4	120.4	C20—C21—H21	119.4
C4—C5—C6	120.42 (17)	C16—C21—H21	119.4
C4—C5—S1	119.85 (14)	N2—C22—C24	113.86 (14)
C6—C5—S1	119.54 (14)	N2—C22—C23	101.65 (13)
C5—C6—C7	118.97 (19)	C24—C22—C23	114.16 (14)
C5—C6—H6	120.5	N2—C22—H22	109.0
C7—C6—H6	120.5	C24—C22—H22	109.0
C2—C7—C6	121.7 (2)	C23—C22—H22	109.0
C2—C7—H7	119.1	O3—C23—N1	125.08 (16)
C6—C7—H7	119.1	O3—C23—C22	128.34 (16)
N2—C8—N1	100.60 (12)	N1—C23—C22	106.56 (14)
N2—C8—C9	110.56 (13)	C29—C24—C25	118.58 (18)
N1—C8—C9	113.73 (13)	C29—C24—C22	122.18 (16)
N2—C8—H8	110.5	C25—C24—C22	119.24 (17)
N1—C8—H8	110.5	C24—C25—C26	120.2 (2)
C9—C8—H8	110.5	C24—C25—H25	119.9
C14—C9—C10	119.34 (17)	C26—C25—H25	119.9
C14—C9—C8	120.91 (16)	C27—C26—C25	120.3 (2)
C10—C9—C8	119.70 (15)	C27—C26—H26	119.8
C11—C10—C9	120.48 (19)	C25—C26—H26	119.8
C11—C10—H10	119.8	C26—C27—C28	120.0 (2)
C9—C10—H10	119.8	C26—C27—H27	120.0
C10—C11—C12	119.5 (2)	C28—C27—H27	120.0
C10—C11—H11	120.3	C27—C28—C29	120.1 (2)
C12—C11—H11	120.3	C27—C28—H28	119.9
C13—C12—C11	120.4 (2)	C29—C28—H28	119.9
C13—C12—H12	119.8	C24—C29—C28	120.8 (2)
C11—C12—H12	119.8	C24—C29—H29	119.6
C12—C13—C14	120.1 (2)	C28—C29—H29	119.6
C12—C13—H13	120.0	C23—N1—C8	111.42 (13)
C14—C13—H13	120.0	C23—N1—S1	122.21 (12)
C9—C14—C13	120.2 (2)	C8—N1—S1	122.10 (11)
C9—C14—H14	119.9	C22—N2—C8	109.39 (12)
C13—C14—H14	119.9	C22—N2—C15	117.95 (12)
N2—C15—C16	116.69 (13)	C8—N2—C15	115.24 (13)
N2—C15—H15A	108.1	O1—S1—O2	120.51 (10)
C16—C15—H15A	108.1	O1—S1—N1	107.54 (8)
N2—C15—H15B	108.1	O2—S1—N1	104.82 (8)
C16—C15—H15B	108.1	O1—S1—C5	108.26 (9)
H15A—C15—H15B	107.3	O2—S1—C5	108.96 (8)
C17—C16—C21	117.71 (17)	N1—S1—C5	105.81 (7)
C17—C16—C15	122.02 (16)		
			
C7—C2—C3—C4	−0.6 (3)	C23—C22—C24—C25	−91.4 (2)
C1—C2—C3—C4	179.1 (2)	C29—C24—C25—C26	−0.4 (3)
C2—C3—C4—C5	0.3 (3)	C22—C24—C25—C26	179.6 (2)
C3—C4—C5—C6	−0.3 (3)	C24—C25—C26—C27	0.0 (4)
C3—C4—C5—S1	174.68 (16)	C25—C26—C27—C28	0.3 (4)
C4—C5—C6—C7	0.5 (3)	C26—C27—C28—C29	−0.1 (4)
S1—C5—C6—C7	−174.51 (15)	C25—C24—C29—C28	0.5 (3)
C3—C2—C7—C6	0.8 (3)	C22—C24—C29—C28	−179.43 (19)
C1—C2—C7—C6	−178.9 (2)	C27—C28—C29—C24	−0.3 (4)
C5—C6—C7—C2	−0.7 (3)	O3—C23—N1—C8	−178.77 (16)
N2—C8—C9—C14	−126.54 (18)	C22—C23—N1—C8	2.63 (18)
N1—C8—C9—C14	121.16 (18)	O3—C23—N1—S1	24.0 (2)
N2—C8—C9—C10	51.0 (2)	C22—C23—N1—S1	−154.56 (11)
N1—C8—C9—C10	−61.3 (2)	N2—C8—N1—C23	16.44 (17)
C14—C9—C10—C11	−1.6 (3)	C9—C8—N1—C23	134.65 (15)
C8—C9—C10—C11	−179.11 (19)	N2—C8—N1—S1	173.67 (10)
C9—C10—C11—C12	0.3 (3)	C9—C8—N1—S1	−68.13 (17)
C10—C11—C12—C13	1.3 (4)	C24—C22—N2—C8	155.71 (13)
C11—C12—C13—C14	−1.7 (4)	C23—C22—N2—C8	32.47 (16)
C10—C9—C14—C13	1.2 (3)	C24—C22—N2—C15	−69.98 (18)
C8—C9—C14—C13	178.72 (19)	C23—C22—N2—C15	166.78 (13)
C12—C13—C14—C9	0.4 (4)	N1—C8—N2—C22	−30.57 (15)
N2—C15—C16—C17	−92.39 (19)	C9—C8—N2—C22	−151.07 (13)
N2—C15—C16—C21	89.38 (19)	N1—C8—N2—C15	−166.24 (13)
C21—C16—C17—C18	−0.4 (3)	C9—C8—N2—C15	73.27 (17)
C15—C16—C17—C18	−178.65 (17)	C16—C15—N2—C22	−56.7 (2)
C16—C17—C18—C19	0.5 (3)	C16—C15—N2—C8	75.00 (18)
C17—C18—C19—C20	−0.6 (3)	C23—N1—S1—O1	37.22 (16)
C18—C19—C20—C21	0.6 (3)	C8—N1—S1—O1	−117.56 (14)
C19—C20—C21—C16	−0.5 (3)	C23—N1—S1—O2	166.60 (13)
C17—C16—C21—C20	0.4 (3)	C8—N1—S1—O2	11.81 (15)
C15—C16—C21—C20	178.67 (16)	C23—N1—S1—C5	−78.31 (15)
N2—C22—C23—O3	160.73 (18)	C8—N1—S1—C5	126.91 (13)
C24—C22—C23—O3	37.7 (2)	C4—C5—S1—O1	−15.47 (17)
N2—C22—C23—N1	−20.73 (16)	C6—C5—S1—O1	159.54 (14)
C24—C22—C23—N1	−143.77 (15)	C4—C5—S1—O2	−148.21 (15)
N2—C22—C24—C29	−27.6 (2)	C6—C5—S1—O2	26.80 (17)
C23—C22—C24—C29	88.6 (2)	C4—C5—S1—N1	99.56 (15)
N2—C22—C24—C25	152.48 (17)	C6—C5—S1—N1	−85.43 (15)
